# Interstrain differences in the expression and activity of Cyp2a5 in the mouse liver

**DOI:** 10.1186/s13104-017-2435-x

**Published:** 2017-03-15

**Authors:** Katia S. Poça, Thiago E. M. Parente, Lucas F. Chagas, Bruna S. Leal, Hellen S. Leal, Francisco J. R. Paumgartten, Ana C. A. X. De-Oliveira

**Affiliations:** Laboratory of Environmental Toxicology, Department of Biological Sciences, National School of Public Health, Oswaldo Cruz Foundation, FIOCRUZ, Av. Brasil 4036, Rio de Janeiro, RJ 21040-361 Brazil

**Keywords:** Cyp2a4, Cyp2a5, Heme oxygenase, Liver toxicity, Coumarin 7-hydroxylase

## Abstract

**Background:**

Cytochrome P450 2A5 (Cyp2a5), a mouse enzyme orthologous of human CYP2A6, catalyzes a number of toxicologically important reactions, including the metabolism of nicotine, aflatoxin B1, and several other xeno- and endobiotics. Cyp2a5 expression is complex and not yet fully understood. We investigated inter-strain differences in the activity and mRNA expression of hepatic Cyp2a5. Cyp1a1/2 and Cyp2b9/10 activities were evaluated for comparative purposes. Data on the interstrain differences in the expression and activity of Cyp2a5 are important to select a suitable mouse model for studying CYP2A6-mediated metabolism.

**Results:**

Activity of Cyp2a5 (coumarin 7-hydroxylase) was highest in DBA-2 and DBA-1, intermediate in B6D2F1 (hybrid) and low in the remaining strains (C57BL/6, C57BL/10, CBA, BALB/cAn, SW). Contrasting with the activity, background levels of Cyp2a4/5 mRNA did not differ between high- and low-activity murine strains. Phenobarbital (PB, 80 mg/kg body weight/day × 3 days, i.p.) increased Cyp2a5, Cyp1a1/2 (ethoxyresorufin-*O*-deethylase) and Cyp2b9/10 (bezyloxyresorufin-*O*-debenzylase) activities while only Cyp2a5 was enhanced by pyrazole (PYR, 100 mg/kg body weight/day × 3 days, i.p.). Inductions of Cyp2a5 activity by PYR and PB were accompanied by increases of Cyp2a4/5 mRNA. PYR and PB did not upregulate heme oxygenase-1 (hmox-1) mRNA expression in any strain, a finding that is apparently at odds with the notion that Cyp2a5 and hmox-1 inductions are coordinated events.

**Conclusions:**

Since background levels of *Cyp2a4/5* gene transcripts of high-activity strains did not differ from those of low-activity mice, distinct constitutive activities did not result from different transcription rates and/or mRNA half-lives. Results therefore suggested that interstrain differences in constitutive activity of Cyp2a5 possibly arise from distinct translation efficiencies, protein half-lives and/or enzyme kinetics toward the substrate. Data from this study indicated that all tested strains are suitable models for studying toxicants that are substrates for human CYP2A6; DBA-2, DBA-1 and the hybrid B62DF1, however, have the advantage of presenting high constitutive activities of Cyp2a5.

**Electronic supplementary material:**

The online version of this article (doi:10.1186/s13104-017-2435-x) contains supplementary material, which is available to authorized users.

## Background

The cytochrome P450 2A gene subfamily (*CYP2A*) encompasses 23 genes and pseudogenes, including four genes identified in mice (*Cyp2a4*, *Cyp2a5*, *Cyp2a12* and *Cyp2a22*), three in rats (*CYP2A1*, *CYP2A2* and *CYP2A3*) and three in humans (*CYP2A6*, *CYP2A7* and *CYP2A13*) [1]. Mouse Cyp2a5 is orthologous of rat CYP2A3 and human CYP2A6. Among all members of CYP2A subfamily, human CYP2A6 (and also CYP2A13) and mouse Cyp2a5 are most similar regarding tissue distribution and substrate specificity. Both CYP2A6 and Cyp2a5 are expressed in the olfactory mucosa, other tissues of the respiratory tract, oesophagus and the liver. Moreover, Cyp2a5 shares many pharmaco- and toxicologically important substrates with CYP2A6 including drugs, coumarin, nicotine, cotinine, aflatoxin B1, the nicotine-derived nitrosamine ketone (NNK), N-nitrosodiethylamine, other xenobiotics and some endogenous compounds (steroid hormones, heme and bilirubin) [2, 3]. *Cyp2a5* gene is expressed in the liver and in extra-hepatic tissues (e.g., olfactory mucosa, kidneys, lungs, brain, small intestines), and its activity and expression in the liver is female-predominant [2, 3].

Regulation of Cyp2a5/CYP2A6 expression is complex and not yet fully understood. *Cyp2a5* is induced by a variety of structurally unrelated chemicals (e.g., metals, pyrazole, phenobarbital) and it can be either up- or down-regulated by pathophysiological conditions such as infections, liver cancer and inflammatory stimuli [2, 4–9]. Hypotheses have been advanced on the involvement of liver injury, oxidative and endoplasmic reticulum stress and perturbations of heme homeostasis in the over-expression of liver *Cyp2a5*/CYP2A6. The mode by which liver pathological conditions regulate Cyp2a5/CYP2A6 expression and activity, however, remains to be elucidated.

Strain differences in the constitutive activity of coumarin 7-hydroxylase (COH, a marker for Cyp2a5 activity) and coumarin metabolism have been reported and the activity recorded in the DBA-2 mice is generally higher than that found in other strains [10–12]. Mouse COH activity has an additive mode of inheritance due to the presence of two alleles at the *Cyp2a5* gene locus on chromosome 7, one for high activity and the other for low activity [10, 13, 14].

This study was designed to investigate interstrain differences in the constitutive activities of Cyp2a5, Cyp1a1/2 and Cyp2b9/10, and Cyp2a5 mRNA expression in the mouse liver. To the authors’ knowledge, no previous study has investigated differences between murine strains in the expression of *Cyp2a4* or *Cyp2a5* genes in the liver. Additionally, we investigated whether the up-regulation of Cyp2a5 activity and expression by known inducers (pyrazole and phenobarbital) was necessarily associated with clinical manifestations of liver damage and enhanced expression of heme oxygenase-1 (*hmox*-*1*). Data from this study are expected to be of help for selection of a suitable mouse model in toxicological studies dealing with xenobiotic compounds metabolized by Cyp2a5/CYP2A6.

## Methods

### Animals

Female mice, 8–10 weeks old, from the Oswaldo Cruz Foundation (FIOCRUZ) breeding stock (Swiss Webster, BALB/cAn, C57BL/6, C57BL/10, CBA, DBA-1, DBA-2, and the F1 hybrid of C57BL/6 female and DBA-2 male, B6D2F1) were used. Six mice of the same strain were housed per cage (standard plastic cage with stainless steel cover lids) and white wood shavings were used as bedding. The animals were maintained under controlled environmental conditions (12 h light/12 h dark cycle, lights on from 7 am to 7 pm; room temperature of 23 ± 2 °C and relative humidity of approx. 70%) with free access to a commercial rodent diet (Nuvital CR1, Nuvilab^®^, Curitiba, PR, Brazil) and filtered tap water. The research project was approved by the “*Ethics Committee on the Use of Animals of the Oswaldo Cruz Foundation*” (CEUA-FIOCRUZ). All procedures were conducted in accordance with Brazilian animal protection and welfare legislation and international guidelines [15].

### Chemicals

Benzyloxy-, ethoxyresorufin, coumarin, EDTA, pyrazole, Bradford reagent, BSA, β-NADP, glucose-6-phosphate, glucose-6-phosphate dehydrogenase, resorufin, umbelliferone and glycine were from Sigma Chemical Co (St. Louis, MO, USA). Phenobarbital (Fenocris^®^) was from Cristália Produtos Químicos Farmacêuticos LTDA (São Paulo, Brazil). All other chemicals used in the experiments were of high analytical grade.

### Treatment

Mice received intraperitoneal (i.p.) injections of pyrazole (PYR, 100 mg/kg body weight/day), phenobarbital (PB, 80 mg/kg body weight/day) or phosphate buffered saline solution only (vehicle-control group, 10 mL/kg body weight/day) for 3 consecutive days, and were euthanized by cervical dislocation 24 h after the last injection. Blood was taken from the retro orbital sinus immediately before the cervical dislocation. Animals were always treated and killed between noon and 2:00 pm. Mice (N = 18 of each strain) were allocated at random (N = 6 per group) to one of the treatment groups (control, PYR and PB).

### Preparation of liver microsomes

After euthanasia, livers were quickly removed, freed from fat and extra tissue, weighed and frozen in liquid nitrogen. Liver microsomal fraction (LMF) was prepared essentially as described by De-Oliveira et al. [16], except for using Tris (100 mM)-KCl (150 mM) buffer (pH 7.4) instead of sucrose solution. LMF was aliquotted in cryogenic tubes that were stored in liquid nitrogen until further use. Protein concentration of LMF was determined by the method of Bradford [17] adapted to a multi-well plate spectrophotometer reader (Spectramax Plus^®^, Molecular Devices, USA).

### Enzyme activities

#### Coumarin 7-hydroxylase

Coumarin 7-hydroxylase activity (COH, a marker for Cyp2a5-catalyzed activity) was assayed essentially as reported by van Iersel et al. [11] with a few modifications: assay tubes (final volume of 0.5 mL) contained 50 mM Tris buffer pH 7.4, 10 µM coumarin and 0.8 mg/mL of protein. After a 3 min pre-incubation of coumarin and microsomal protein, the reaction was initiated by addition of a NADPH regenerating system (0.5 mM β-NADP, 10 mM glucose 6-phosphate, 0.5 U/mL glucose 6-phosphate dehydrogenase and 10 mM magnesium chloride). Reaction was carried out for 10 min at 37 °C with shaking until being stopped by the addition of 2 N HCl to assay tubes. The reaction product, umbelliferone, was taken to tubes containing a 1.6 M glycine-NaOH (pH 10.4) solution and transferred to quartz cuvettes for fluorescence measurement in a spectrofluorimeter (Shimadzu RF5301PC). The equipment parameters were set as follows: excitation at 355 nm, emission at 460 nm and band slit width at 3 nm. A standard curve of umbelliferone was run in parallel with each assay.

#### Alkoxy-resorufin-*O*-dealkylases

Benzyloxy- (Cyp2b9/10) and ethoxy- (Cyp1a1/2) resorufin-*O*-dealkylases (BROD and EROD, respectively) were assayed in 96-well microplates as described by Kennedy and Jones [18] with some modifications. The final concentrations of components in the reaction were 5 µM substrate (benzyloxy- or ethoxi-resorufin), 0.25 mM β-NADP, 5 mM glucose 6-phosphate, 0.5 U/mL glucose 6-phosphate dehydrogenase and 2.5 mM magnesium chloride. A constant amount of microsomal protein (0.025 mg) was added to each well. After a 10 min reaction time at 37 °C in a shaker water-bath, acetonitrile was added to each well. The product of the reaction (resorufin) was measured using a fluorescence plate reader (Spectramax Gemini XS^®^, Molecular Devices, USA) with excitation and emission wavelengths set at 530 nm and 590 nm, respectively.

#### Alanine and aspartate aminotransferases

Serum alanine (ALT) and aspartate aminotransferase (AST) activities were determined by a colorimetric method using a commercially available kit (Bioclin^®^, Belo Horizonte, MG, Brazil) adapted to a multi-well plate spectrophotometer reader (Spectramax Plus^®^, Molecular Devices, USA), and absorbance was registered at 505 nm.

### Determination of mRNA levels

mRNA was extracted from the liver tissue with TRI Reagent^®^ and quantified using a Nanodrop^®^ spectrophotometer while cDNA was synthesized using the High Capacity RNA-to-cDNA kit^®^ (Applied Biosystems^®^) and a T100™ thermocycler (BioRad^®^). TaqMan^®^ gene expression assays were purchased from Applied Biosystems^®^ (Mm00487248_g1 for *Cyp2a4/5*, Mm00516007_m1 for *hmox*-*1* and 4352341E for *β*-*actin*, used as endogenous control). Real-time reactions were performed in a Step One Plus real-time thermocycler (Applied Biosystems^®^). The relative quantification of the target genes was made using the Q-Gene software application (Equation 3 of the manuscript) [19].

### Statistical analysis

Data following a normal distribution (e.g. enzyme activities) were analyzed by one-way analysis of variance (ANOVA) and Dunnett’s post hoc test. For data that are known not to be normally distributed (e.g., percentages and ratios), the Kruskal–Wallis test was used, followed by the Mann–Whitney *U* test with Bonferroni correction. Statistical evaluation was performed using GraphPad Prism version 5.01 for Windows (GraphPad Software, San Diego, California USA), and differences were considered statistically significant at a value of P < 0.05.

## Results and discussion

### Interstrain differences in Cyp2a5 activity

**Fig. 1:**
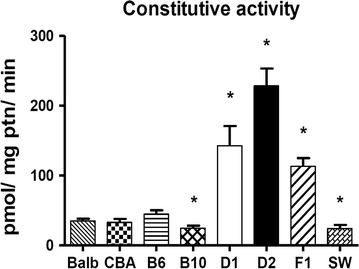
Constitutive coumarin-7-hydroxylase activity (pmoles umbelliferone/mg ptn/min) in liver microsomes of different strains of mice. Histogram bar heights are mean ± SEM. Strain COH activities that differ from that of B6 strain are indicated by an *asterisk* above the *bar* (*P < 0.05, ANOVA and Dunnett’s post hoc test). N = 6 per mouse strain

Results from this study showed that constitutive (non-induced) activity of COH in the liver microsomal fraction markedly varied among mouse strains (P < 0.05, ANOVA and Dunnett’s post hoc test). The DBA-2 (D2) strain—the oldest of all inbred strains of mice—ranked first for COH activity (mean ± SEM: 228.4 ± 24.8 pmol/mg ptn/min), while DBA-1 (D1) (142.5 ± 28.3 pmol/mg ptn/min) exhibited the second highest activity. Of the remaining murine strains, CBA, BALB/cAn (BALB) and C57BL/6 (B6) presented COH activities ranging from 32.9 ± 5.0 to 44.5 ± 6.0 pmol/mg ptn/min, while the lowest activities were found in C57BL/10 (B10) (24.4 ± 3.8 pmol/mg ptn/min) and Swiss Webster (SW) mice (24.1 ± 5.3 pmol/mg ptn/min). Owing to its additive mode of inheritance, Cyp2a5 (COH) activity in the liver of the hybrid B6D2F1 (F1) (113.3 ± 11.6 pmol/mg ptn/min) was in between those of its parental high- (D2) and low- (B6) activity strains (Fig. 1; Table 1).

**Table 1:** Liver Cyp2a5 (COH), Cyp1a1/2 (EROD) and Cyp2b9/10 (BROD) activities in different mouse strains. Female mice were treated ip with PBS (CON), pyrazole (PYR 100 mg/kg bw/days × 3 days), or phenobarbital (PB, 80 mg/kg bw/days × 3 days) and euthanized 24-h after the last dose

Treatment	Monooxygenase activity (pmoles/mg protein/min)								
	COH			EROD			BROD		
	CON	PYR	PB	CON	PYR	PB	CON	PYR	PB
Mouse strain									
BALB/cAn	35.2 ± 2.9	69.7 ± 4.4*	75.3 ± 6.0*	71.2 ± 6.0	103.1 ± 9.0	532.7 ± 77.5*	56.6 ± 5.7	53.3 ± 4.0	192.8 ± 23.9*
CBA	32.9 ± 5.0	119.7 ± 5.2*	91.9 ± 5.4*	74.9 ± 4.9	61.5 ± 5.5	293.0 ± 33.7*	22.0 ± 2.3	19.8 ± 3.5	212.5 ± 22.4*
C57BL/6	44.5 ± 6.0	112.1 ± 8.4*	62.2 ± 5.5	99.2 ± 7.2	98.7 ± 8.6	444.3 ± 28.6*	47.3 ± 5.8	33.4 ± 4.8	158.9 ± 8.7*
C57BL/10	24.4 ± 3.8	88.1 ± 4.1*	63.6 ± 5.9*	132.2 ± 9.4	135.5 ± 16.9	598.7 ± 59.5*	42.6 ± 3.5	72.9 ± 29.9	181.3 ± 5.5*
DBA-1	142.5 ± 28.3	344.8 ± 50.6*	261.8 ± 24.6	50.2 ± 3.3	50.0 ± 4.0	265.7 ± 13.1*	26.8 ± 1.4	43.4 ± 12.4	271.0 ± 2.3*
DBA-2	228.4 ± 24.8	507.7 ± 12.5*	493.5 ± 11.7*	74.9 ± 7.9	64.3 ± 6.3	338.1 ± 14.8*	39.8 ± 2.8	41.0 ± 3.8	266.9 ± 28.5*
B6D2F1	113.3 ± 11.6	437.8 ± 24.3*	308.4 ± 20.6*	68.8 ± 3.4	66.6 ± 3.6	251.1 ± 14.1*	35.2 ± 4.1	45.0 ± 5.3	306.5 ± 8.8*
SW	24.1 ± 5.3	78.5 ± 8.8*	42.5 ± 3.1	72.8 ± 7.6	74.8 ± 9.1	348.5 ± 29.7*	41.2 ± 7.8	38.8 ± 10.2	143.6 ± 4.4*

Values are shown as mean ± SEM. Data were evaluated by ANOVA and Dunnett’s post hoc test. Means that are different (P < 0.05) from respective controls are indicated by an asterisk (*). N = 6 mice of each strain per group (CON, PYR or PB)

*COH* (coumarin 7-hydroxylase) pmoles umbelliferone/mg protein/min, *EROD* (ethoxyresorufin-), *BROD* (benzyloxy-resorufin-*O*-dealkylase) pmoles resorufin/mg protein/min

PYR (100 mg/kg body weight/day i.p. × 3 days) enhanced COH activity in all strains (Table 1) with induction factors (IF: ratio of induced to non-induced activity) ranging from 2 to 2.5-fold for BALB, D2, D1 and B6 mice, and from 3.3 to 3.9-fold for SW, CBA, B10 and F1 mice (see Additional file 1). The administration of PB (80 mg/kg body weight/day i.p. × 3 days), a pleiotropic inducer of xenobiotic biotransformation enzymes, increased COH activity in all strains, except B6, D1 and SW (Table 1). The induction of COH by PB, however, was less marked than that caused by PYR. Induction factors (IFs) after treatment with PB ranged from 2.1 to 2.8-fold for BALB, D2, B10, F1 and CBA mice (see Additional file 1).

In summary, results indicated that COH, a Cyp2a5-mediated activity, varies up to 10-fold between mouse strains. Notwithstanding the marked variation of constitutive activities, mice from all strains evaluated in this study responded to challenges with PB and PYR, a finding that suggests that constitutive strain-related differences do not involve xenoreceptors or mechanisms by which Cyp2a5-mediated activity is induced by xenobiotics. It is of note that PB, a pleiotropic inducer of mice Cyp2a5, 2b9/10, 3a11 and other CYP isoforms, and PYR, an inducer of Cyp2a4, 2a5, 2e1 and 2j, enhance Cyp2a5-catalyzed activities by distinct mechanisms [20–22].

### Interstrain differences in Cyp1a and Cyp2b activities

Strain-related variations of Cyp1a1/2 (EROD) and Cyp2b9/10 (BROD) constitutive activities were far less pronounced than those observed with Cyp2a5 (COH) activity, and ratios of the highest to the lowest strain activity were 2.6 and 2.5-fold for Cyp1a1/2 (B10 = 132.2 ± 9.4 pmol/mg ptn/min; D1 = 50.2 ± 3.3 pmol/mg ptn/min) and Cyp2b9/10 (BALB = 56.6 ± 5.7 pmol/mg ptn/min; CBA = 22.0 ± 2.3 pmol/mg ptn/min), respectively (Table 1).

PYR did not enhance Cyp1a1/2- and Cyp2b9/10-mediated activities in any mouse strain (i.e., IFs were nearly 1), a finding that is consistent with the notion that this heterocyclic diazole compound is a selective inducer of Cyp2a, 2j and Cyp2e1 activities in the liver tissue [22].

PB, on the other side, markedly enhanced activities mediated by Cyp1a1/2 and Cyp2b9/10 in all mouse strains, thereby confirming that it causes a pleiotropic induction of hepatic monooxygenase activities. Treatment with PB provoked a nearly fourfold increase in Cyp1a1/2 activity (EROD) in all mouse strains, except for BALB, the IF of which was 7.5 (see Additional file 2). As far as induction of Cyp2b9/10 (BROD) is concerned, the effect of PB on CBA (9.7 fold), D1 (10.1 fold), F1 (8.7-fold) and D2 (6.7 fold) was more pronounced than the effect on BALB, B6, B10 and SW (3 < IF < 5) (see Additional file 3). Chatuphonprasert et al. [23] also noted that inductions of EROD and BROD by PB in D2 were stronger than the inductions in B6 mice.

### Interstrain differences in *Cyp2a4/5* expression

**Fig. 2:**
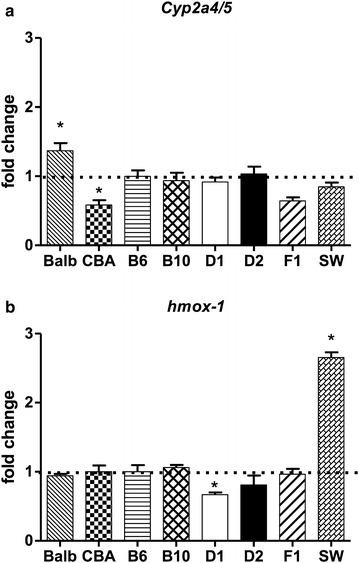
Constitutive levels of Cyp2a4/5 and hmox-1 mRNA in the liver of mice from different strains. **a** (*upper panel*) expression of *Cyp2a4/5*. **b** (*lower panel*) expression of *hmox*-*1*. Relative quantification of mRNA was made by qPCR taking C57BL/6 liver sample as the reference (*P < 0.05, Kruskal–Wallis test followed by Mann–Whitney U test with Bonferroni’s correction). N = 6 per mouse strain

**Fig. 3:**
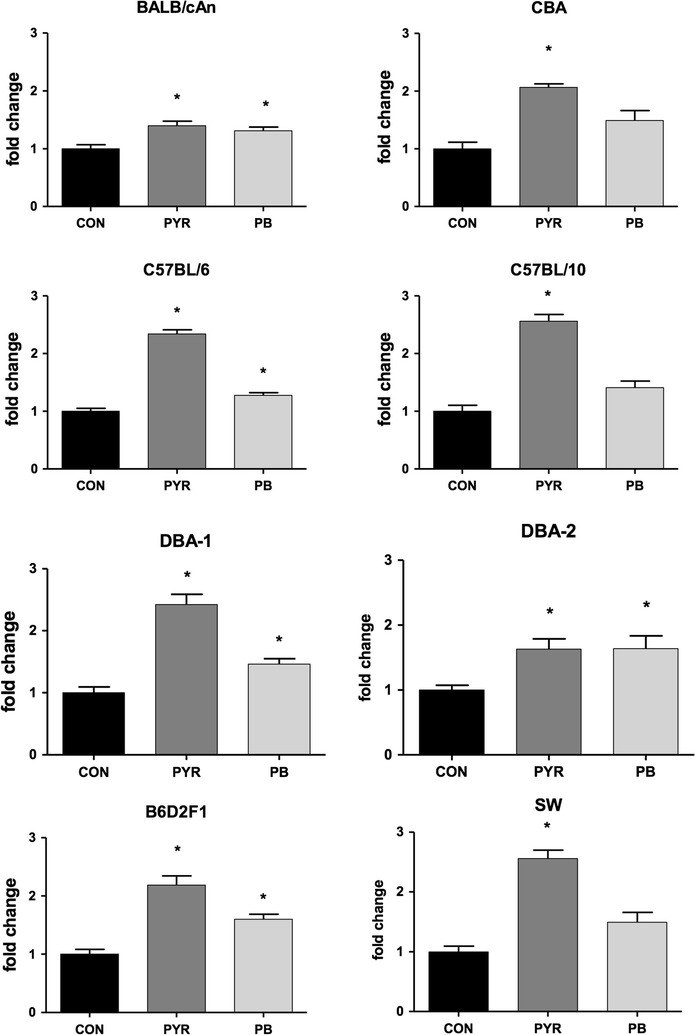
Pyrazole- and phenobarbital-induced expression of liver Cyp2a4/5 mRNAs in mice from different strains. Female mice were treated with phosphate buffered saline (CON, PBS 10 mL/kg body weight/day × 3 days, i.p), pyrazole (PYR, 100 mg/kg body weight/day × 3 days, i.p.) or phenobarbital (PB, 80 mg/kg body weight/day × 3 days, i.p.). Relative quantification of mRNA was made by qPCR taking the control mice (CON) as the reference. An *asterisk* (*) above the *bar* indicates that mRNA levels differ (P < 0.05, Kruskal–Wallis test followed by Mann–Whitney U test with Bonferroni’s correction) from those of vehicle-controls (CON) of the same strain. N = 6 mice of each strain per treatment group

*Cyp2a4* gene is highly related to *Cyp2a5* (>98% identity in the amino acid sequences of their coding region) and both are expressed in the mouse liver. Despite their high degree of similarity, *Cyp2a4* and *Cyp2a5* genes code for enzymes that have distinct substrate specificities: while the former mediates 15-α-hydroxylation of steroid hormones (e.g., testosterone and estrogens), the latter catalyzes the 7-hydroxylation of coumarin. A single amino acid substitution (Phe209Leu) seems to be sufficient to convert the specificity of Cyp2a5 reaction from coumarin 7-hydroxylase to testosterone 15-α-hydroxylase [24]. Primers and probe used in this study do not distinguish between the highly similar transcripts of *Cyp2a4* and *Cyp2a5*, so that levels of both mRNAs were quantified jointly. As shown in Fig. 2, non-induced levels of Cyp2a4/5 mRNAs exhibited only minor inter-strain differences (Kruskal–Wallis test followed by Mann–Whitney U test, P < 0.05 when levels were compared to B6), a result that is at odds with the pronounced strain differences in constitutive levels of Cyp2a5–mediated activities (COH) (Table 1). For instance, while constitutive activity of COH in D2 mice was nearly 10-fold that found in SW, Cyp2a4/5 mRNA levels did not differ between the two strains. The induction of Cyp2a5 activity (COH) by PYR, however, was accompanied by an elevation of Cyp2a4/5 mRNA levels in all mouse strains (Fig. 3). The effect of PB on Cyp2a4/5 mRNA was less evident than that of PYR. Although having enhanced Cyp2a4/5 expression in BALB/c, B6, D1, D2 and F1 mice, PB did not produce statistically significant elevations of Cyp2a4/5 mRNA over constitutive levels in CBA, B10 or SW strains (Fig. 3).

Using a PCR-enhanced diagnostic analysis based on Squires and Negishi’s method [25], Hahnemann et al. [26] estimated the relative contributions of Cyp2a4 and Cyp2a5 to the total Cyp2a4/5 mRNA content in the liver of D2 and B6 male mice. The authors found that in both mouse strains, Cyp2a5 mRNA represents nearly 90% of constitutive levels of total Cyp2a4/5 mRNA. Hahnemann et al. [26] also suggested that increases in the total Cyp2a4/5 mRNA produced by PYR were predominantly due to elevations in Cyp2a5 mRNA levels. This interpretation is also corroborated by data showing that PYR enhanced COH activity (Cyp2a5-mediated), but did not alter 15-α-hydroxylase activity (Cyp2a4-mediated) in the D2 mice [26].

A number of studies demonstrated that liver Cyp2a5 can be induced by a myriad of structurally unrelated organic and inorganic chemicals such as PYR, PB, CCl_4_, cocaine, griseofulvin, thioacetamide, metals such as cadmium, indium and cobalt and also by some infectious diseases such as hepatitis, malaria and fascioliasis [8, 9, 27, 28]. The mechanisms by which chemical agents and infections induce Cyp2a5 are not entirely understood. It is generally accepted that induction of liver Cyp2a5 activity and mRNA levels involves both transcriptional and post-transcriptional events [29]. The exact role played by several transcription factors (nuclear receptors) in the regulation of *Cyp2a5* expression, however, is not completely elucidated.

Results presented here showed that PYR increased levels of total Cyp2a4/5 mRNA in all strains (Fig. 3), a finding that is consistent with those reported by other authors for D2 and B6 mice treated with this heterocyclic diazole compound [26, 30]. It was described that the half-life of Cyp2a5 mRNA in the liver of PYR-treated D2 mice was at least fourfold longer than that in untreated controls, an indication that increases in *Cyp2a5* gene transcript levels were predominantly due to mRNA stabilization [30]. Subsequent studies revealed that PYR increases the level of a heterogeneous nuclear riboprotein A1 (hnRNPA1) that binds to a 71-nucleotide region of Cyp2a5 mRNA, thereby protecting it from degradation [20, 31, 32]. These studies strongly suggested that induction of liver Cyp2a5 by PYR is a mainly post-transcriptional and pre-translational event that involves Cyp2a5 mRNA stabilization. The mechanism by which PYR and related compounds produce alterations of Cyp2a5 mRNA-binding proteins, however, is not entirely understood.

The induction of Cyp2a5 by PB, on the other side, seems to involve enhanced translational efficiency and/or protein stabilization in addition to increased transcription rates. For instance, while increasing Cyp2a protein and Cyp2a5 activity (COH), PB did not change levels of Cyp2a4/5 mRNA in the liver of male D2 mice [30]. Nonetheless, Hahnemann et al. [26] reported that PB enhanced Cyp2a4/5 mRNA and Cyp2a5 activity by 2- to 3-fold in the liver of C57BL/6 mice. In the present study, while increasing Cyp2a5 activity (COH) by 2- to 3-fold in most strains (Table 1), PB produced only a slight (<twofold) increase in Cyp2a4/5 mRNA levels (Fig. 3).

The most striking finding of the interstrain comparison was that, while presenting much higher constitutive activities of Cyp2a5 (Table 1), D2 and D1 (and also the hybrid B6D2F1) did not exhibit levels of Cyp2a4/5 mRNA more elevated than those found in the remaining mouse strains (Fig. 2).

It is known that some xenobiotic metabolizing enzyme mRNAs do not correlate well with protein expression and consequently with enzyme activities. Chang et al. [33], for instance, found that CYP1A2 mRNA and protein (immunoblot analysis) levels were not correlated in human liver samples. Along the same line, Ohtsuki et al. [34] reported that human liver CYP1A2, CYP2A6, CYP2C9, CYP2E1 and CYP4A11 correlated poorly whereas other CYPs correlated highly (CYP2B6, CYP2C8, CYP3A4) or moderately (CYP2C9, CYP2D6, CYP3A5, CYP3A7) with protein (quantified by LC/MS/MS analysis) expression levels. Ohtsuki et al. [34] also noted that, for most CYPs, there was a better correlation of enzyme activities to protein levels than to mRNAs levels. The reasons why mRNAs of some human liver CYPs including CYP2A6 did not correlate well with protein expression are unclear.

In principle, increased enzyme activity when mRNAs levels remain unchanged could be explained either because of protein stabilization (which increases protein levels) or due to post-translational/allosteric mechanisms (which do not alter protein levels). Since we did not quantify protein levels in the present study, it was not possible to exclude one of these two explanations for the inter-strain differences in the constitutive activity of Cyp2a5. The interpretation that inter-strain differences in Cyp2a5 activity might result from differences in degree of protein stabilization (and protein levels), however, is consistent with enzyme kinetic data provided by other studies. Although COH V_max_ determined in liver microsomes from D2 was higher than that measured in microsomes from AKR and C57BL/6, the reaction K_m_ did not differ either between D2 and AKR [35], or between D2 and B6 strains [36]. Since K_m_ was essentially the same, strain distinct COH constitutive activities should result from different amounts of Cyp2a5 protein in liver microsomes. As far as the authors are aware, no previous study has compared the constitutive levels of Cyp2a4/5 (or Cyp2a5) mRNAs between mouse strains using a quantitative real-time PCR method (qPCR). The lack of quantitative data on the Cyp2a5 protein expression levels in the evaluated mouse strains, however, is an important limitation of this study.

### Hepatotoxicity and Cyp2a5 induction

**Fig. 4:**
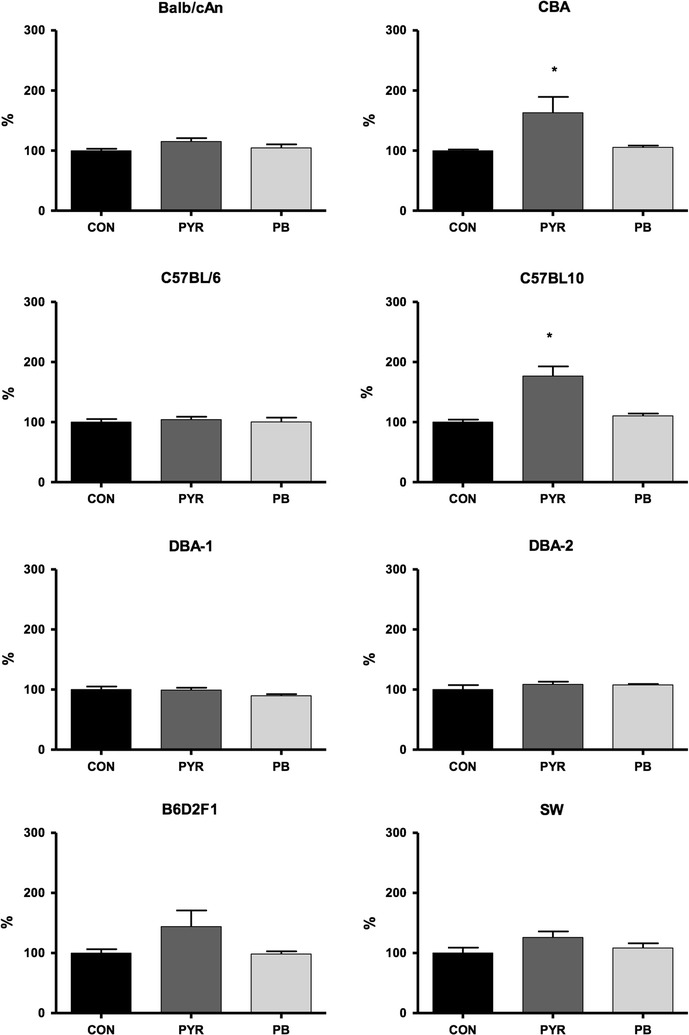
Interstrain differences in serum levels of ALT after treatment with pyrazole or phenobarbital. ALT serum levels (IU/L) in BALB/c, CBA, B6, B10, D1, D2, F1 and SW mice treated with phosphate buffered saline (CON, PBS 10 mL/kg body weight/day × 3 days, i.p), pyrazole- (PYR, 100 mg/kg body weight/day × 3 days, i.p.) or phenobarbital (PB, 80 mg/kg body weight/day × 3 days, i.p.). ALT serum levels are expressed as ratio of PYR- or PB-treated to average control group levels (100%). * levels are different from those of vehicle controls (CON) of the same strain (P < 0.05, Kruskal–Wallis test followed by Mann–Whitney U test with Bonferroni’s correction). N = 6 mice of each strain per treatment group

Since Cyp2a5 expression and activity are up-regulated by hepatotoxic agents and some liver pathological conditions [8, 9], it was postulated that oxidative stress or another pathophysiological change associated with liver injury may eventually elicit *Cyp2a5* overexpression [6, 37]. PYR is hepatotoxic and dose regimens of this heterocyclic compound that cause strong inductions of Cyp2a5 also produce a marked elevation of serum aminotransferases (ALT and AST), a marker for liver damage, as demonstrated after treatment with one single injection of 200 mg/kg bw/days [6], 150 mg/kg bw/days × 2 days [38] or one single injection of 100 mg/kg bw of PYR [6]. In the present study, statistical analysis (Kruskal–Wallis test followed by Mann–Whitney U test, P < 0.05) showed a modest (<twofold) increase of ALT in CBA (IF = 1.6) and B10 (IF = 1.8) treated with PYR. ALT serum levels remained unaltered in PB-treated mice (Fig. 4). AST was not altered in PYR treated mice. Except for a small elevation (<twofold) of AST serum levels in B10 and D2 mice, AST was not altered by administration of PB either (Additional file 4). Therefore, the dose regimen of PYR (100 mg/kg bw/days × 3 days) chosen for this study was sufficient to up-regulate Cyp2a5 expression and activity without causing clinically evident liver injury.

### Concomitant induction of *hmox*-*1* and *Cyp2a4*/*5* expression

Some authors postulated that all pathophysiological conditions and hepatotoxic chemicals that up-regulate Cyp2a5 share the common feature of altering cellular redox state in the liver tissue [37, 39, 40]. Along this line, it was suggested that Nrf2, a transcription factor regulated at the post-transcription level by oxidative stress, plays a key role in the induction of Cyp2a5 by agents and conditions that eventually lead to liver damage. Nfr2 also mediates the expression of a set of redox homeostasis genes including *hmox*-*1* [40] and concomitant inductions of liver Cyp2a5 and hmox-1 in D2 mice intoxicated with Cd [41] and in those infected with malaria parasites [9] were reported. Enhanced hmox-1 activity results in increased production of bilirubin, the accumulation of which is potentially toxic. If Cyp2a5 is in fact involved in the oxidation of bilirubin, as suggested by Abu-Bakar et al. [41], a concurrent up-regulation of hmox-1 and Cyp2a5 in some toxic and pathological conditions, by maintaining a balance between bilirubin production and elimination, would confer a certain protection against liver damage caused by enhanced oxidative stress.

**Fig. 5:**
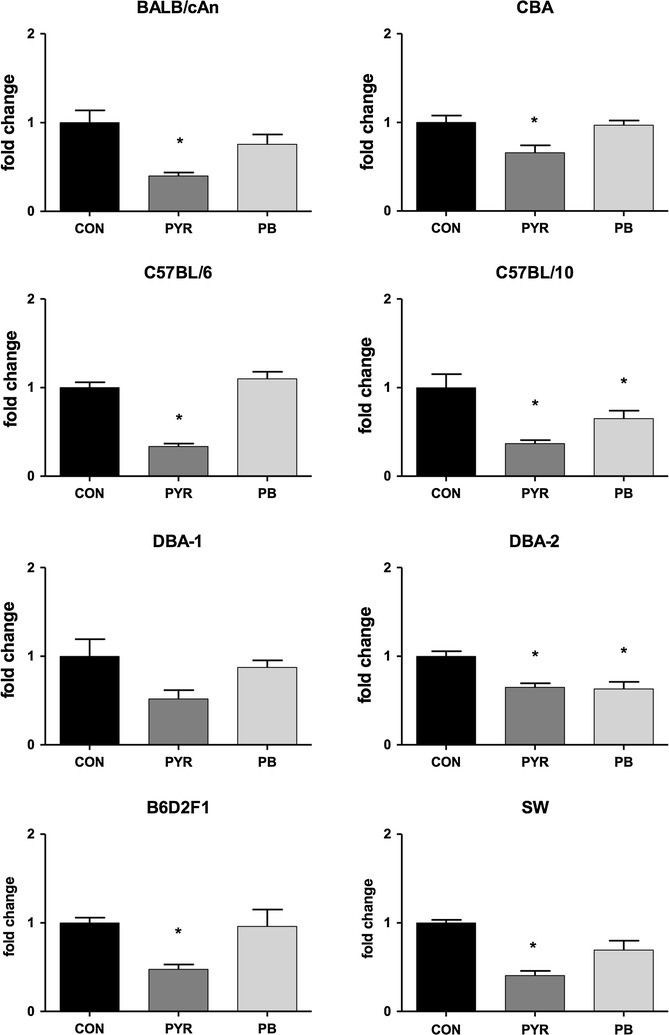
Pyrazole and phenobarbital induced expression of liver hmox-1 mRNAs in mice from different strains. Female mice were treated with phosphate buffered saline (CON, PBS 10 mL/kg body weight/day × 3 days, i.p), pyrazole (PYR, 100 mg/kg body weight/day × 3 days, i.p.) or phenobarbital (PB, 80 mg/kg body weight/day × 3 days, i.p.). Relative quantification of mRNA was made by qPCR taking the control mice (CON) as the reference. An *asterisk* (*) above the *bar* indicates that mRNA levels differ (P < 0.05, Kruskal–Wallis test followed by Mann–Whitney U test with Bonferroni’s correction) from those of vehicle-controls (CON) of the same strain. N = 6 mice of each strain per treatment group

Data from this study, however, showed that PYR, at a dose regimen that induced Cyp2a5 activity (and Cyp2a4/5 mRNA expression) in the absence of signs of hepatotoxicity, failed to enhance *hmox*-*1* expression in the livers of BALB, CBA, B6, B10, D2, F1 and SW mice (Fig. 5). Nichols and Kirby [42] found an enhanced expression of *hmox*-*1* (micro-array analysis confirmed with q-RT-PCR) in the liver of B6 mice 24-h after treatment with a high dose of PYR (200 mg/kg bw, ip). Unlike the PYR dose regimen used in our study (100 mg/kg bw/d ays × 3 days), the PYR dose employed by Nichols and Kirby [42] induced a marked rise in ALT serum levels.

Differences in the response of hmox-1 and Cyp2a5 to treatment with PbCl_2_ and MeHg were observed in mouse primary hepatocytes [40]. Although Pb and Hg activated Nfr2 and enhanced both *hmox*-*1* and *Cyp2a5* expression, the up-regulation of *hmox*-*1* gene was more rapid and transient as compared to that of *Cyp2a5*. Moreover, contrasting with a lack of induction of *Cyp2a5* expression, an enhanced expression of *hmox*-*1* was still present in hepatocytes from Nfr2 null mice [40]. According to the authors, a possible explanation for the aforementioned differences could be an involvement of the transcriptional repressor Bach 1 in the regulation of *hmox*-*1* gene. In other words, while Nfr2 would be critical for regulating Cyp2a5 in any case, inactivation of Bach 1 would induce hmox-1 in the Nfr2 null mouse.

## Conclusions

In conclusion, results from this study showed that constitutive activity of Cyp2a5 (COH) in the liver of D2 and D1 were clearly higher than that of the outbred SW or the inbred strains BALB, CBA, B6 and B10. As expected from an additive mode of inheritance, Cyp2a5 activity of the hybrid B6D2F1 was intermediate between those of its high- and low-activity parents (DBA-2 and C57BL/6, respectively). Since background levels of *Cyp2a4/5* gene transcripts of high-activity strains (D1, D2) did not differ from those of low-activity mice (e.g., SW, B6, B10), distinct constitutive activities did not result from different transcription rates and/or mRNA half-lives. The absence of data on Cyp2a5 protein levels was an important limitation of this study. Owing to the lack of quantitative data on protein expression levels it was not possible to elucidate whether constitutive activities of Cyp2a5 correlated with protein levels. Differences in protein stabilization and/or in post-translational/allosteric mechanisms are possible explanations for strain differences in Cyp2a5 constitutive activity.

It was also shown that PYR up-regulated Cyp2a5 activity and *Cyp2a4/5* expression, but did not affect Cyp1a1/2 and Cyp2b9/10 activities in the liver of mice from any strain. As expected from a pleiotropic inductor, PB increased activities of Cyp2a5, Cyp1a1/2 and Cyp2b9/10 as well. In CBA, B10 and SW PB-caused induction of Cyp2a5 activity was unaccompanied by a clear enhancement of Cyp2a4/5 mRNA levels, a finding that is consistent with the prevailing notion that PB-mediated CYP2A induction involves mainly actions at translational and/or post-translational levels (Fig. 3). Finally, Cyp2a5 up-regulation by PYR, a known hepatotoxin, was unaccompanied by clinical signs of liver toxicity with the dose used in this study, a result that differs from the results observed by Gilmore et al. [6]. In this study, PYR and PB did not up-regulate hmox-1 mRNA, a finding that is at odds with the idea that Cyp2a5 and hmox-1 inductions are associated events that share a common Nfr2-activation pathway.

## Additional files

**Additional file 1.** Inter-strain differences in the induction of liver COH activity by pyrazole and phenobarbital.

**Additional file 2.** Inter-strain differences in the liver EROD activity.

**Additional file 3.** Inter-strain differences in the liver BROD activity.

**Additional file 4.** AST in the serum of mice treated with PYR or PB.

## Abbreviations

ALT: alanine aminotransferase
AST: aspartate aminotransferases
BROD: benzyloxyresorufin-*O*-debenzylase
bw: body weight
COH: coumarin 7-hydroxylase
EROD: ethoxyresorufin-*O*-deethylase
hmox-1: heme oxygenase 1
IF: induction factor
LC/MS/MS: liquid chromatography/tandem mass spectrometry
LMF: liver microsomal fraction
Mouse strains: D1, DBA-1; D2, DBA-2; B6, C57BL/6; B10, C57BL/10; F1, F1 hybrid of C57BL/6 female and DBA-2-male
SW: Swiss Webster
NfR2: nuclear factor (erythroid-derived 2)-like 2 (also know as NEFE2L2)
PB: phenobarbital
PYR: pyrazole
qPCR: real-time polymerase chain reaction
