# Supplementary material for: Interstrain differences in the expression and activity of Cyp2a5 in the mouse liver
Source: BMC Res Notes. 2017 Mar 15;10:125. doi: 10.1186/s13104-017-2435-x (PMC5353797; doi:10.1186/s13104-017-2435-x)
Supplement: Supplementary file 1 — Additional file 1. Inter-strain differences in the induction of liver COH activity by pyrazole and phenobarbital. [file 13104_2017_2435_MOESM1_ESM.pdf]

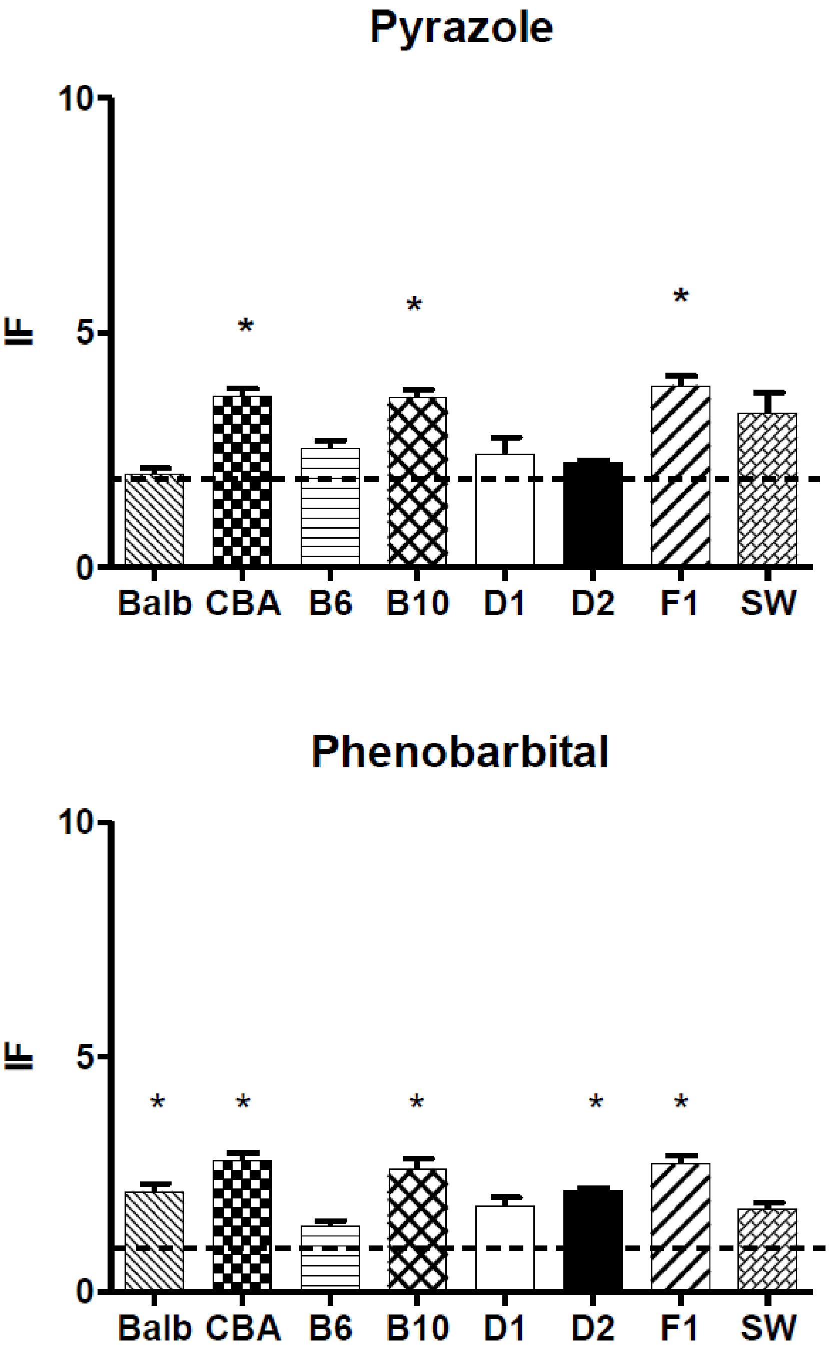

Additional file 1. Induced Coumarin-7-hydroxylase activities in liver microsomes of different strains of mice. (upper panel) Induced activities: Induction factor (IF, ratio of induced to average constitutive activity) after treatment with pyrazole (100 mg/kg body weight/day x 3 days, i.p.) and (lower panel) Induced activities: Induction factor (IF, ratio of induced to average constitutive activity) after treatment with phenobarbital (80 mg/kg body weight/day x 3 days, i.p.). * differs from B6 ($P<0.05$, Kruskal-Wallis test followed by Mann-Whitney U test with Bonferroni's correction).
