# Supplementary material for: Interstrain differences in the expression and activity of Cyp2a5 in the mouse liver
Source: BMC Res Notes. 2017 Mar 15;10:125. doi: 10.1186/s13104-017-2435-x (PMC5353797; doi:10.1186/s13104-017-2435-x)
Supplement: Supplementary file 2 — Additional file 2. Inter-strain differences in the liver EROD activity. [file 13104_2017_2435_MOESM2_ESM.pdf]

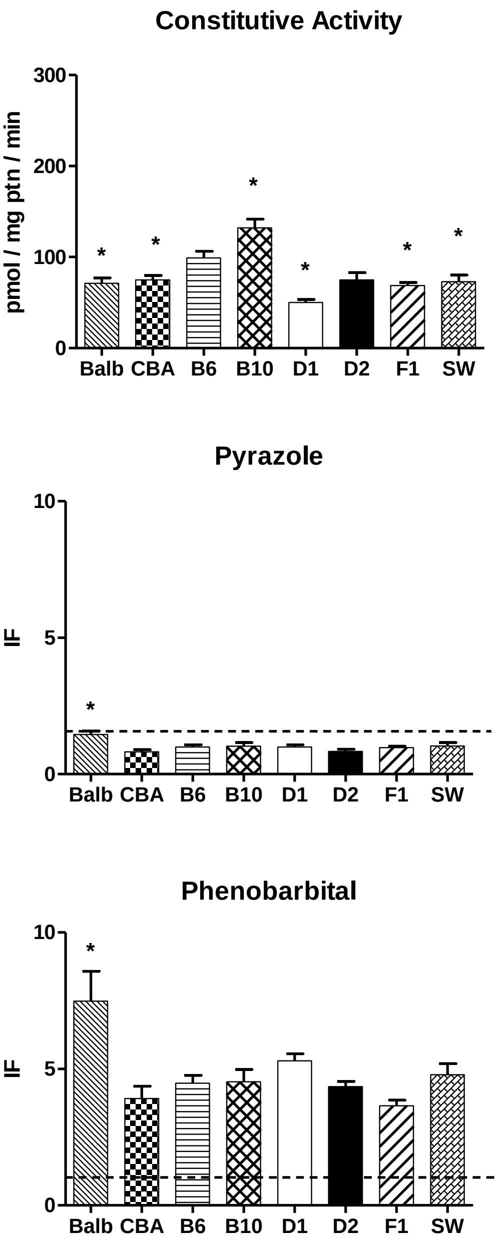

Additional file 2. Constitutive and induced ethoxy-resorufin-*O*-deethylase activities in liver microsomes of different strains of mice. (upper panel) Constitutive ethoxy-resorufin-*O*-deethylase (EROD) activities (pmoles resorufin/mg ptn/min); (middle panel) Induced activities: Induction factor (IF, ratio of induced to average constitutive activity) after treatment with pyrazole (100 mg/kg body weight/day x 3 days, i.p.) and (lower panel) Induced activities: Induction factor (IF, ratio of induced to average constitutive activity) after treatment with phenobarbital (80 mg/kg body weight/day x 3 days, i.p.) *: differs from B6 (P<0.05, Kruskal Wallis test followed by Mann-Whitney U test).
