# Supplementary material for: Interstrain differences in the expression and activity of Cyp2a5 in the mouse liver
Source: BMC Res Notes. 2017 Mar 15;10:125. doi: 10.1186/s13104-017-2435-x (PMC5353797; doi:10.1186/s13104-017-2435-x)
Supplement: Supplementary file 3 — Additional file 3. Inter-strain differences in the liver BROD activity. [file 13104_2017_2435_MOESM3_ESM.pdf]

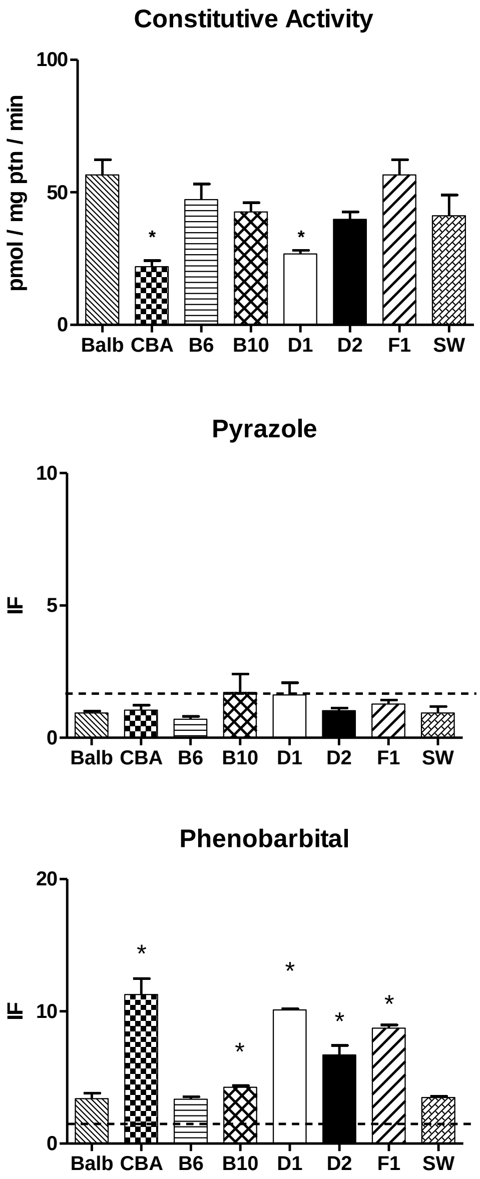

Additional file 3. Constitutive and induced benziloxy-resorufin-*O*-debenzylase activities in liver microsomes of different strains of mice. (upper panel) Constitutive benziloxy-resorufin-*O*-debenzylase (BROD) activities (pmoles resorufin/mg ptn/min); (middle panel) Induced activities: Induction factor (IF, ratio of induced to average constitutive activity) after treatment with pyrazole (100 mg/kg body weight/day x 3 days, i.p.) and (lower panel) Induced activities: Induction factor ((IF, ratio of induced to average constitutive activity) after treatment with phenobarbital (PB: 80 mg/kg body weight/ day x 3 days, i.p.) *: differs from B6 ($P<0.05$, Kruskal Wallis test followed by Mann-Whitney U test).
