# Supplementary material for: Interstrain differences in the expression and activity of Cyp2a5 in the mouse liver
Source: BMC Res Notes. 2017 Mar 15;10:125. doi: 10.1186/s13104-017-2435-x (PMC5353797; doi:10.1186/s13104-017-2435-x)
Supplement: Supplementary file 4 — Additional file 4. AST in the serum of mice treated with PYR or PB. [file 13104_2017_2435_MOESM4_ESM.pdf]

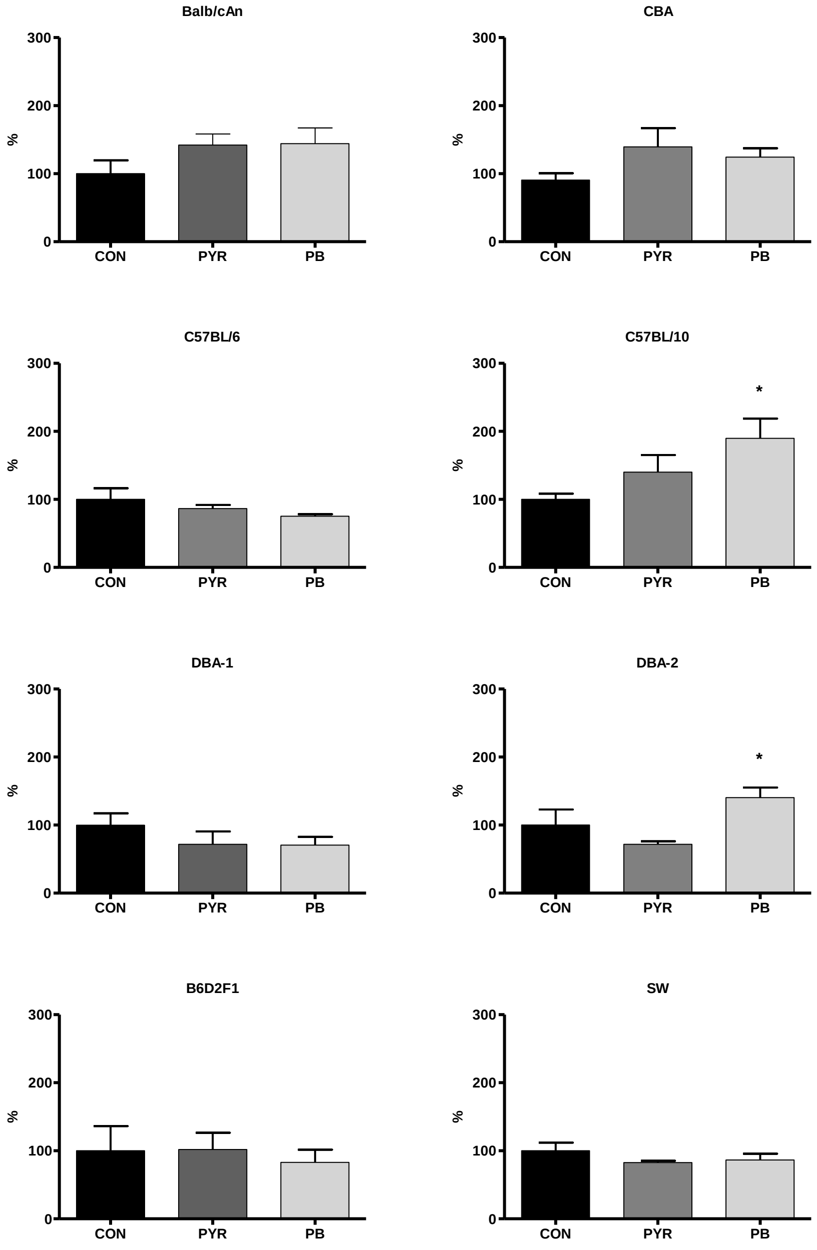

Additional file 4. Serum transaminase levels in different strains of mice. Serum AST levels in sera of non-treated, pyrazole- (100 mg/kg body weight/day x 3 days, i.p.) and phenobarbital- (80 mg/kg body weight/day x 3 days, i.p.) treated Balb, CBA, B6, B10, D1, D2, F1 and SW mice. Levels (IU/L) are expressed as ratio of induced to average constitutive levels (100%). *: levels are different from those of vehicle controls of the same strain (P<0.05, Kruskal Wallis test followed by Mann-Whitney U test).
